# A Short Review on Various Engineering Applications of Electrospun One-Dimensional Metal Oxides

**DOI:** 10.3390/ma14185139

**Published:** 2021-09-07

**Authors:** Weronika Smok, Tomasz Tański

**Affiliations:** Department of Engineering Materials and Biomaterials, Faculty of Mechanical Engineering, Silesian University of Technology, 44-100 Gliwice, Poland; tomasz.tanski@polsl.pl

**Keywords:** electrospinning, metal oxides, nanomaterials, nanofibers, nanowires

## Abstract

The growing scientific interest in one-dimensional (1D) nanostructures based on metal-oxide semiconductors (MOS) resulted in the analysis of their structure, properties and fabrication methods being the subject of many research projects and publications all over the world, including in Poland. The application of the method of electrospinning with subsequent calcination for the production of these materials is currently very popular, which results from its simplicity and the possibility to control the properties of the obtained materials. The growing trend of industrial application of electrospun 1D MOS and the progress in modern technologies of nanomaterials properties investigations indicate the necessity to maintain the high level of research and development activities related to the structure and properties analysis of low-dimensional nanomaterials. Therefore, this review perfectly fits both the global trends and is a summary of many years of research work in the field of electrospinning carried out in many research units, especially in the Department of Engineering Materials and Biomaterials of the Faculty of Mechanical Engineering and Technology of Silesian University of Technology, as well as an announcement of further activities in this field.

## 1. Introduction

**Figure 1:**
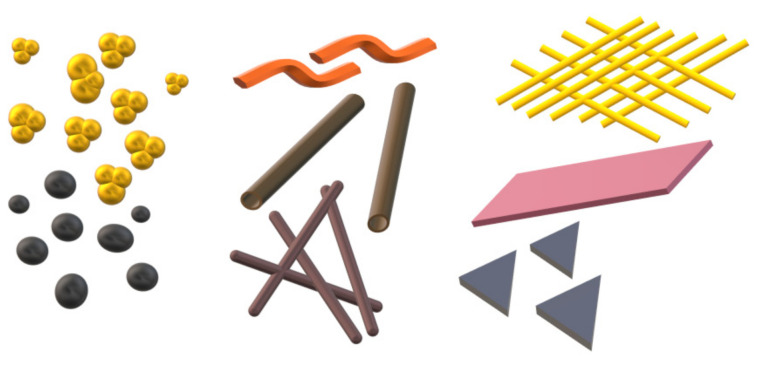
Nanomaterials classification: zero-dimensional, one-dimensional, two-dimensional [4,5].

One of the main streams of materials engineering in Poland and worldwide, on which the attention of not only the scientific community but also the industry is currently focused, are nanomaterials and methods of their fabrication. This interest is not unfounded because nanomaterials exhibit much more favorable mechanical properties compared to those presented by traditional materials and outstanding physicochemical properties due to their large specific surface area and quantum effects observed at the nanometer scale [1,2,3]. Nanomaterials in terms of the number of dimensions that remain below 100 nm can be classified as follows: zero-dimensional (0D), one-dimensional (1D), two-dimensional (2D) and three-dimensional (3D) (Figure 1) [4,5]. 

**Figure 2:**
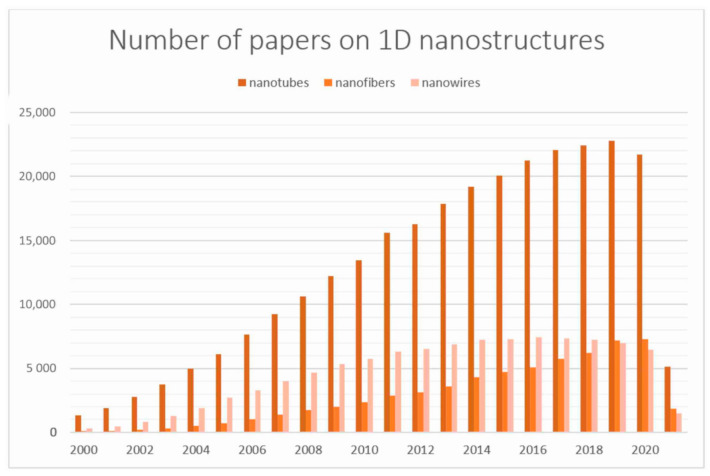
Number of papers on one-dimensional nanostructures in 2000–2021 (keywords: nanotubes, nanofibers, nanowires, Scopus database).

Each of the above-mentioned groups of materials has a huge application potential in almost all industrial fields, especially in those that require a constant search for new solutions and technologies to ensure a high quality of manufactured products. However, in the last two decades, one-dimensional nanoobjects seem to have stood out in popularity over the other types of nanomaterials, which is due to their quantum-confined structure determining unique optical, electrical, magnetic and thermoelectric properties [6]. This feature makes it possible to obtain the desired properties by controlling the size of the nanostructure [7,8,9]. The number of publications shows that the most commonly fabricated and studied one-dimensional nanomaterials for three decades continuously included nanotubes, especially carbon nanotubes, while 1D nanostructures in the form of nanofibers and nanowires have definitely been less popular so far (Figure 2). Not only worldwide but also in Poland, the great potential of 1D nanomaterials is recognized, which can be inferred by analyzing data from the Polish National Science Center (NCN), which indicate that over the past decade, NCN has funded over 400 projects in this area and currently 12 such studies are being completed.

**Figure 3:**
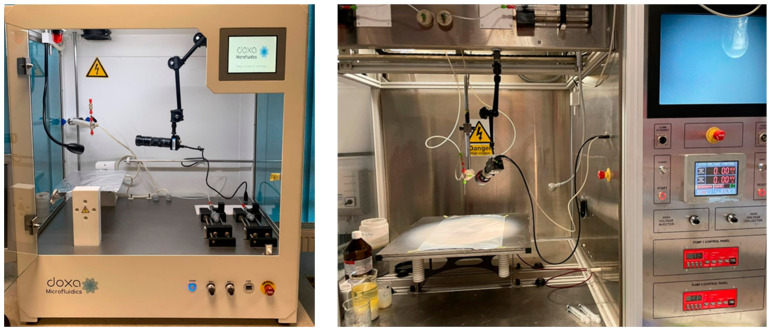
Electrospinning devices used in the laboratory of the Department of Engineering Materials and Biomaterials at the Silesian University of Technology.

Due to the strongest quantum effect among various one-dimensional nanomaterials, it is metal oxide semiconductor nanostructures that are most often studied for applications in the development of modern solar cells, optoelectronic and acoustic devices, liquid crystal devices and detectors. To date, researchers have devoted the most attention to ZnO, TiO_2_, SiO_2_ and Bi_2_O_3_. However, there are increasing publications on other 1D metal oxides, including In_2_O_3_ and SnO_2_ [10,11,12,13,14,15,16,17,18,19,20,21,22,23,24,25,26,27,28,29,30]. According to recent scientific reports, these types of materials represent the future of semiconductor-based devices, so it is important to focus on their development and on selecting the most advantageous method for their fabrication. To date, many methods have been developed for the fabrication of one-dimensional nanomaterials, including chemical and physical vapor deposition methods (CVD and PVD), salt and hydrothermal methods, controlled growth from liquid phase (VLS), matrix synthesis, nanolithography or electrospinning from solution [15,20,31,32,33,34,35,36,37,38,39,40,41,42,43,44,45,46,47,48,49,50,51,52,53,54]. Nevertheless, the last mentioned method has a particular advantage over the others. Electrospinning allows the production of materials not only on a laboratory scale, but also on an industrial scale through the modifications, such as use of multineedle or needle-less processes. Moreover, it does not require a complicated apparatus (Figure 3) or expensive precursors, and allows the control of the morphology and properties of the obtained products with only a few parameters. It is worth noting that, unlike PVD and CVD methods, this technique also does not require a protective atmosphere to manufacture uncontaminated, pure nanomaterial. Thus, the nanostructures obtained by this method are ready for use without further functionalization or purification. Electrospinning in combination with subsequent high-temperature processing enables producing high quality oxide one-dimensional nanostructures in an uncomplicated way with the desired properties, among others [10,11,31,35,48,51,55,56,57,58,59,60,61,62,63].

The great importance of the electrospinning method in the manufacturing of one-dimensional nanomaterials of all types is confirmed by numerous projects carried out in Polish research units and financed by the National Science Center. According to information obtained from the NCN website, 11 out of 24 projects using electrospinning as the main method of manufacturing research objects are currently being carried out (Table 1). The majority of grants awarded concerned the potential application of 1D nanostructures in medicine, while the remaining ones concerned photovoltaics, catalysis and purification of the water environment. The popularity of this method in Poland is also confirmed by the number of publications originating from Polish institutions. According to the Scopus database, more than 500 Polish scientific articles have been published to date, most of which come from the Department of Engineering Materials and Biomaterials (T. Tański and W. Matysiak in cooperation with W. Smok, M. Zaborowska and P. Jarka), the Institute of Physics (T. Błachowicz), the Silesian University of Technology, the Institute of Fundamental Technological Research, the Polish Academy of Sciences (P. Sajkiewicz), the International Center of Electron Microscopy for Material Science, AGH University of Science and Technology (Z.J. Krysiak, U. Stachewicz) and the Materials Design Division, Warsaw University of Technology (W. Święszkowski). These data confirm how high the expectations and hopes are for the development of this manufacturing method.

A research group from the Department of Engineering Materials and Biomaterials is in the process of implementing the NSC project entitled ‘‘New polymer structures for the construction of photovoltaic cells’’ based on the fabrication of nanostructures from ZnO and TiO_2_ [10,59,64,65,66]; in addition, one member of the group is pursuing a Diamond Grant entitled “Hybrid one-dimensional nanostructures X (X = ZnO and/or TiO_2_)-Yb^3+^/Eu^3+^ obtained by hybrid methods with enhanced photocatalytic activity”.

**Table 1:** The 11 projects financed by National Science Centre on one-dimensional nanomaterials that are currently implemented in Polish scientific units.

No.	Project Title	Scientific Unit	Status	Research Object	Ref.
1	The use of collagen for surface functionalization using chemical methods of polycaprolactone nanofibers formed by the electrospinning technique	Institute of Fundamental Technological Research, Polish Academy of Sciences	Current 10 July 2017 09 July 2021	Manufacturing of three types of nanofibers from various aliphatic polysters—poly (caprolactone), poly (L-lactide) and their copolymer and functionalization of their surface.	[67]
2	New polymer structures for the construction of photovoltaic cells	University of Silesia in Katowice, Faculty of Science and Technology, Silesian University of Technology, Faculty of Mechanical Engineering	Current 11 October 2017 10 June 2021	Preparation of composites containing a dispersed phase in the form of a conductive polymer or inorganic ZnO and TiO_2_ nanoparticles or hybrid systems made of these fillers and optical properties analysis.	[68]
3	Innovative biocatalytic systems produced by the immobilization of enzymes on multifunctional materials synthesized by electrospinning	Poznan University of Technology, Faculty of Chemical Technology	Current 01 March 2019 28 February 2022	The use of materials produced by the electrospinning method for the immobilization of selected enzymes of environmental importance and the application of the obtained biocatalytic systems in the processes of dye degradation.	[69]
4	Multifunctional composite materials enriched with natural polyphenols for potential applications in tissue engineering	AGH University of Science and Technology, Faculty of Materials Science and Ceramics	Current 22 October 2018 21 October 2022	Design and production of new, multifunctional, bioresorbable composites enriched with polyphenols (PPh) obtained from medicinal plants (sage/rosemary) and individual polyphenolic compounds (rosmarinic acid and carnosic acid).	[70]
5	Thermosensitive hydrogels filled with bioactive nanofibers for regeneration of neural tissue	Institute of Fundamental Technological Research, Polish Academy of Sciences	Current 21 January 2019 20 January 2022	Design and manufacturing of a smart, injectable hydrogel, loaded with short electrospun, bioactive PLLA and laminin nanofibers for central nervous system tissue engineering.	[71]
6	Cellular responses to the properties of electrospun polymer fibers for tissue engineering applications	AGH University of Science and Technology, International Centre of Electron Microscopy for Materials Science	Current 05 February 2020 04 February 2023	Determining the relationship between the conductive and structural properties of polymer electrospun tissue scaffolds and cell growth for regenerative medicine applications.	[72]
7	Removal of selected environmental pollutants from water solutions with the use of immobilized laccase	Poznan University of Technology, Faculty of Chemical Technology	Current 20 August 2020 30 September 2021	Development of a methodology for the production of new carriers in the form of electrospun nanofibers and membranes in the immobilization of enzymes, and then the use of immobilized enzyme systems in the remediation of phenolic compounds from aqueous solutions.	[73]
8	Nanofibrous mucoadhesive carrier of brinzolamide based on hydroxypropyl cellulose and β-cyclodextrin.	Institute of Fundamental Technological Research, Polish Academy of Sciences	Current 09 July 2020 08 July 2023	Optimization of the chemical composition and production conditions of a modern nanofiber material intended for the gradual local release of an ophthalmic drug.	[74]
9	Investigation of the properties of the nature-inspired polymer nanofiber networks in the context of their application for water recovery and energy generation	AGH University of Science and Technology, Faculty of Materials Science and Ceramics	Current 01 September 2016 28 February 2022	Understanding the process of wetting nanofibers due to their properties and using this knowledge to increase the efficiency of the process of collecting water from the fog, by incorporating nanofibers into the currently used Fog Water Collectors.	[75]
10	Bioactive materials capable of mimicking the state of hypoxia with high osteogenic and angiogenic potential	Jagiellonian University in Kraków, Faculty of Chemistry	Current 02 October 2019 01 October 2022	Production of scaffolding containing particles of bioactive glasses modified with transition metal ions by electrospinning.	[76]
11	Electrical properties and catalytic activity against I-/I3-pair redox reactions of hierarchical carbon nanostructures with a new Ni-Co bimetallic catalyst	AGH University of Science and Technology, Faculty of Materials Science and Ceramics	Current 20 February 2020 19 February 2022	Synthesis of hierarchical composites based on electrospun carbon nanofibers and metallic nanoparticles and their catalytic properties.	[77]

## 2. Electrospinning of Metal Oxides 1D Nanostructures

**Figure 4:**
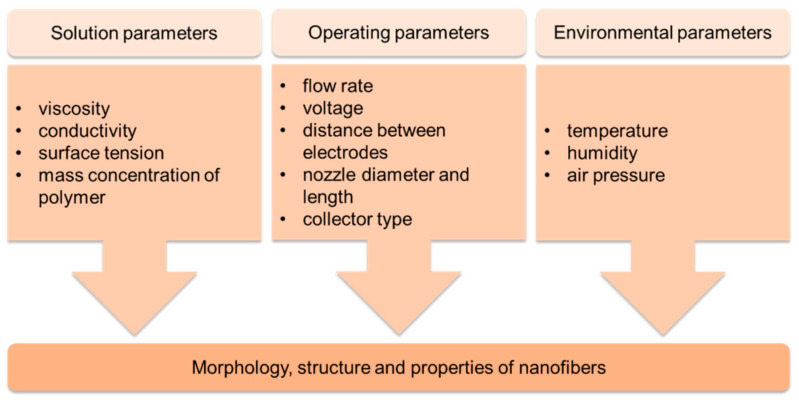
Diagram of electrospinning process parameters.

The electrospinning technique has been known and used for nearly three decades. It involves the use of an electrostatic field created between electrodes (nozzle and collector) under the influence of high voltage to form and stretch a droplet of spinning solution into a fiber settling in a spiral motion on the collector, resulting in a fibrous (nano)mat [78,79,80,81,82,83,84]. The fibers obtained by this method are characterized by their nanometric diameter and considerable length, reaching up to several meters, and their structure, morphology and properties can be controlled by the parameters used, which can be divided into 3 groups (Figure 4) [84].

**Figure 5:**
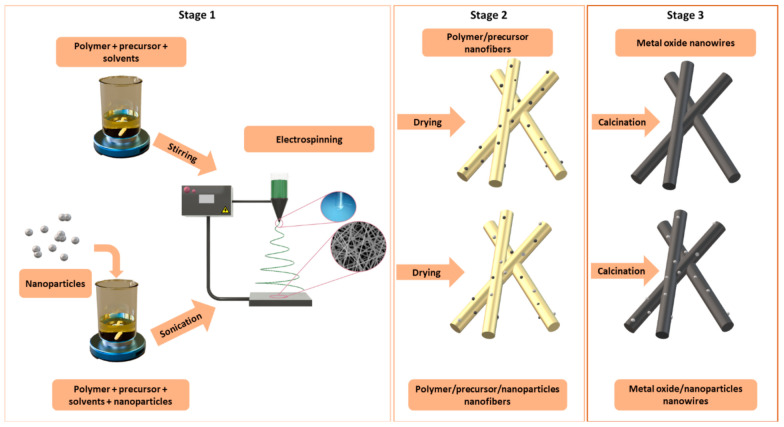
Scheme of metal oxide nanowires formation in 3 main stages: solution preparation, electrospinning and calcination process.

In the case of fabrication of metal oxide-based nanostructures, the electrospinning process is only an intermediate step, followed by temperature treatment (calcination) of the spun nanofibers to remove the polymer matrix. The entire fabrication process is shaped as follows: in the first stage (Stage 1, Figure 5), it is necessary to prepare a spinning solution, containing a given polymer (for each type of experiment, an appropriate polymer is selected each time, for example, it can be polyvinylpyrrolidone (PVP), polyacrylonitrile (PAN) or poly(vinyl alcohol)) (PVA) ensuring appropriate viscosity and precursor molecules (these are most often metal chlorides or nitrates) (Table 2). 

**Figure 6:**
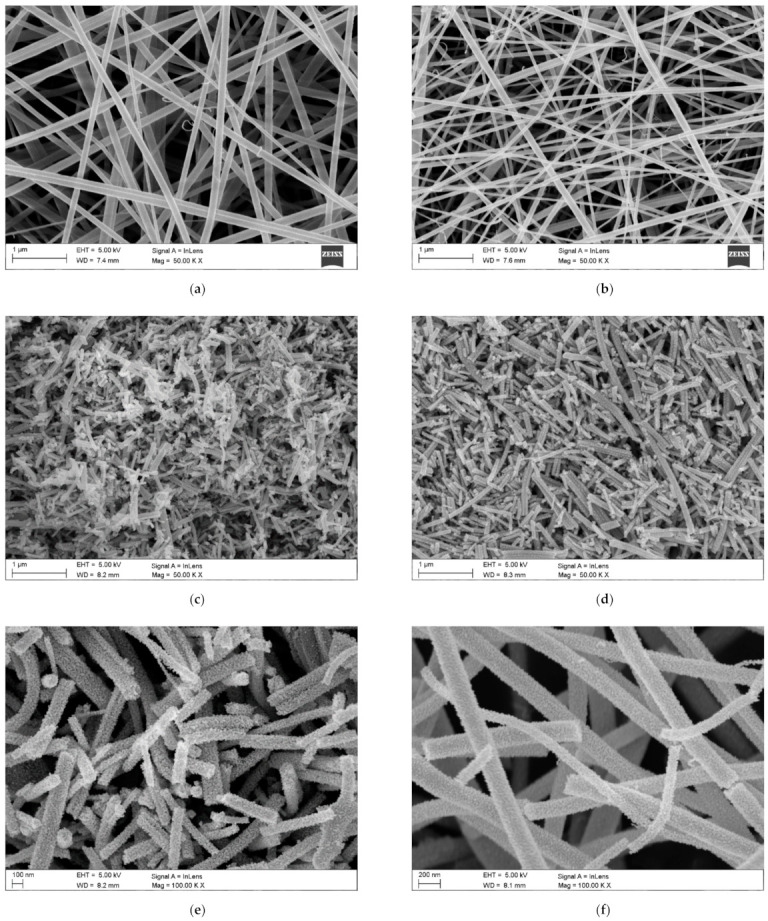

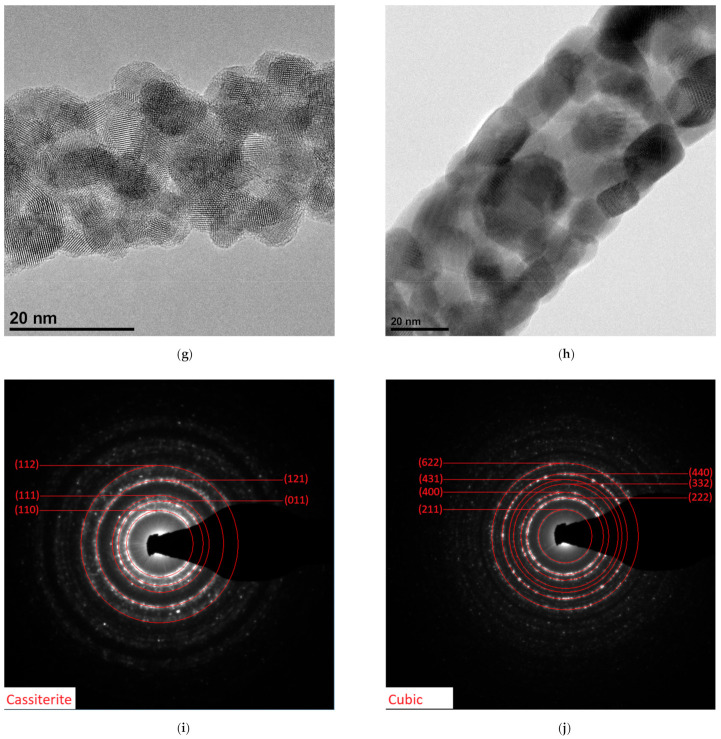
Scanning Electron Microscope (SEM) images of (**a**) PVP/SnCl_4_ nanofibers, (**b**) PVP/In(NO_3_)_3_ nanofibers (Stage 2 Figure 5. (**c**,**e**) SnO_2_ nanowires after calcination in 500 °C, (**d**,**f**) In_2_O_3_ nanowires after calcination in 500 °C (Stage 3 Figure 5), Transmission Electron Microscope (TEM) images of (**g**) SnO_2_, (**h**) In_2_O_3_ single nanowire, Selected Area Electron Diffraction (SAED) patterns of: (**i**) SnO_2_ nanowires and (**j**) In_2_O_3_ nanowires [own study].

Then, the homogeneous solution (the time and temperature of the homogenization process are experimentally chosen for each solution individually) is placed in the device pump from where it is fed through feed channels to the nozzle, where it is subjected to electrostatic field forces to form polymer/precursor composite nanofibers (Stage 2 Figure 5), which takes place due to solvents (e.g., ethanol (EtOH), *N,N*-Dimethyloformamide (DMF)) evaporation. To obtain the final product, which is 1D MOS, the spun polymer-precursor nanofibers are calcined until the organic phase is completely removed and nanostructures based on one or more oxides or with other dopant material are formed (Stage 3 Figure 5). Figure 6 shows the morphology and structure of SnO_2_ and In_2_O_3_ nanowires formed as a result of calcination of polymer/precursor nanofibers (Figure 6a,b) at a temperature of 500 °C, which are the subject of research by the research group from the Department of Engineering Materials and Biomaterials. Smooth, continuous, free from structural defects, polymer/precursor nanofibers (Figure 6a,b) after calcination became discontinuous, polucrystalline nanowires composed of ceramic nanoparticles (Figure 6c–j).

**Figure 7:**
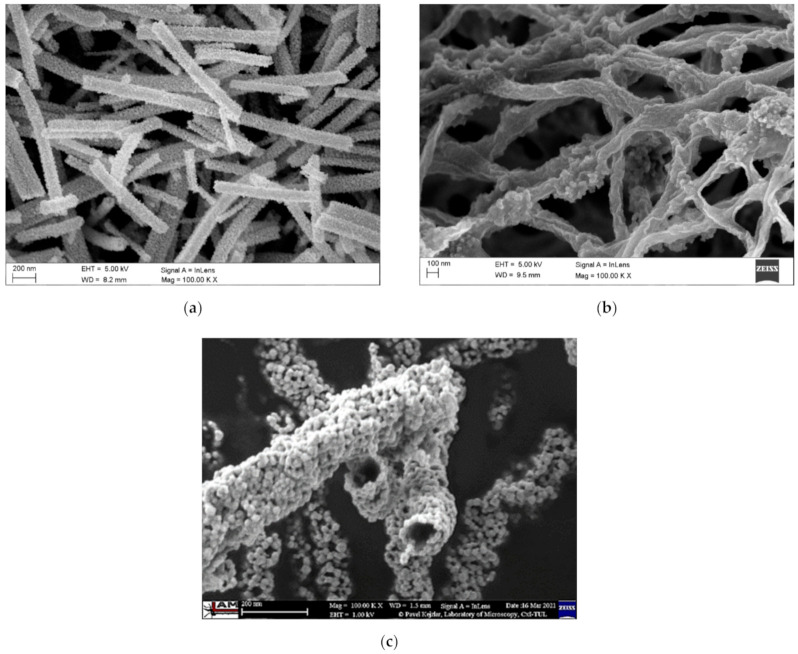
Various 1D morphologies of SnO_2_ nanostructures produced by electrospinning: (**a**) traditional nanowires; (**b**) nanowires decorated with nanoparticles; (**c**) nanotubes [own study].

The calcination parameters having a significant impact on the morphology and structure of nanostructures include time, temperature and atmosphere of the process; depending on them, it is possible to obtain nanomaterials with an amorphous, crystalline and mixed structure, as well as in the form of classical nanowires, decorated nanowires or nanotubes (Figure 7).

Among Polish units, the research on the preparation of nanostructures of various structure, morphology and properties by electrospinning and 1D calcination is being intensively carried out by the research group from the **Department of Engineering Materials and Biomaterials of the Silesian University of Technology** in cooperation with non-Polish centers, e.g., the Center for Nanomaterials, Advanced Technologies and Innovations (Technical University of Liberec), Department of Machines and Apparatus, Electromechanical and Power Systems, Faculty of Engineering Mechanics (Khmelnytskyi National University) and the Department of Physics, Faculty of Electrical Engineering (University of Žilina).

The growing interest in the production and industrial application of one-dimensional MOS-based nanostructures as well as the progress in modern technologies of nanomaterials production and testing indicate the necessity to maintain a high level of research and development activities related to the analysis of morphology, structure and properties of 1D metal oxides. Therefore, this review article perfectly fits in with relevant global trends and is a continuation of many years of research work in the field of nanomaterials produced by electrospinning carried out in the Department of Engineering Materials and Biomaterials of the Faculty of Mechanical Engineering and Technology of the Silesian University of Technology.

**Figure 8:**
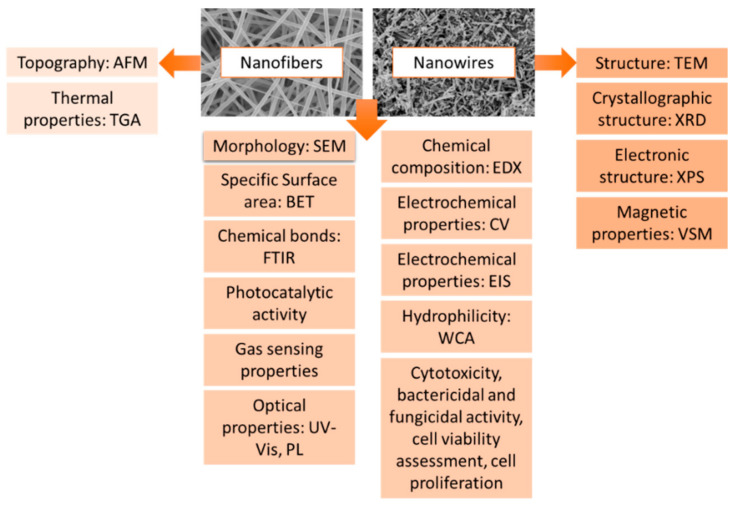
Research methodology of nanomaterials produced by the electrospinning method.

The one-dimensional nanomaterials obtained by electrospinning and calcination are characterized by a unique morphology, structure and properties, which can be investigated by the following methods: Atomic Force Microscopy (AFM), Thermogravimetric Analysis (TGA), SEM, Brunauer–Emmett–Teller surface area analysis (BET), Fourier-transform infrared spectroscopy (FTIR), Photoluminecence (PL), Energy-dispersive X-ray spectroscopy (EDX), cyclic voltammetry (CV), Electrochemical impedance spectroscopy (EIS), Water contact angle (WCA), X-ray diffraction (XRD), X-ray photoelectron spectroscopy (XPS), Vibrating-sample magnetometer (VSM) and TEM. (Figure 8).

The application potential of one-dimensional nanostructures based on metal oxides is very broad and covers areas such as photocatalysis, photovoltaics, energy storage, medicine, opto-electronic devices, microwave absorption and especially gas sensors [99,100,101,102].

## 3. Selected Applications of Electrospun 1D MOS Nanostructures

### 3.1. Electrospun 1D MOS in Saving the Natural Environment

Industrialization and increasing consumerism have led to the highest level of warning about environmental pollution and its associated crisis. Industrial waste compared to municipal waste is toxic and non-biodegradable, as it contains heavy metal ions, oils and fats, dyes, phenols and ammonia, which can adversely affect human life and health but also the environment. One possible solution to this problem is to use the process of photocatalysis to break down harmful substances into simpler and environmentally friendly ones. Photocatalysis combines reactions using light and a catalyst, which is usually a semiconductor—it absorbs light and acts as a catalyst for chemical reactions. Therefore, it is necessary to search for semiconductor materials that can help solve this global problem.

**Figure 9:**
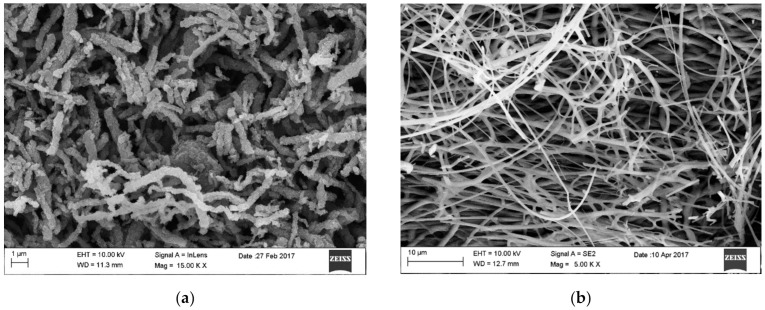
SEM image of surface morphology of (**a**) TiO_2_ nanowires after calcination at 400 °C, (**b**) ZnO nanowires after calcination at 400 °C [own study].

Recently, electrospun one-dimensional semiconductor metal oxide nanostructures, predisposed by their unique optical and electrical properties, have attracted the attention of researchers studying photocatalytic pollutant decomposition processes of TiO_2_, ZnO and SnO_2_, whose energy gap width, radiation absorption range and mobility rate can be controlled by the parameters of the manufacturing process (Figure 9, Table 3 and Table 4) [86,103,104,105,106,107].

**Figure 10:**
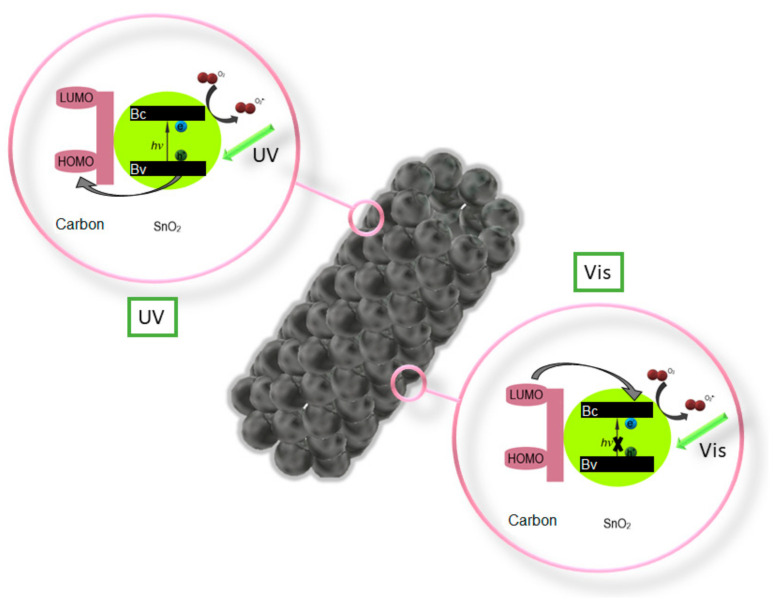
Diagram of the photocatalytic activation of nanotubes SnO_2_ [113].

Z. Wang et al. performed an analysis [111] of the photocatalytic properties of ZnO/SnO_2_ nanofiber with and without the addition of the P123 precursor, which resulted in a much higher photocatalytic activity in the degradation of methyl orange (MO) in UV light of the composite nanofibers with the addition of P123. C. Zhu et al. in their work [112] showed significantly greater possibilities of photocatalytic decomposition of Rhodamine B in visible light through the use of composite SnO_2_/Fe_2_O_3_ nanofibers compared to the capabilities of the non-admixture SnO_2_. Electrospun SnO_2_ nanostructures coated with a 1 nm thick carbon shell fabricated by P. Zhang et al. [113] showed very efficient photocatalytic degradation of 4-Nitrophenol under both UV and visible light (Figure 10). K. Wang et al. in their work [114] reported a study on the photocatalytic activity of mutiheterojunction in the photodegradation of methyl orange (MO) and Cr (VI) ions under visible light. It was observed that the SnO_2_/Bi_2_O_3_/BiOI nanofibers were characterized by better photocatalytic activity than the non-admixture SnO_2_ and Bi_2_O_3_, which the authors attributed to the increased absorption of visible light, electron-hole pair separation and large specific surface area of the nanostructures studied.

T. Wang. et al. [86] demonstrated that the use of magnetic field-assisted electrospinning in the fabrication of nanofibers and nanotubes from TiO_2_ narrowed the band gap to favor photocatalytic performance—TiO_2_ reduced Rhodamine B (RhB) by 95.8% in 100 min. Q. Zhang et al. [115] proposed the use of 1D composite nanostructures based on In_2_O_3_ of admixtured CaIn_2_O_4_ in the photocatalytic purification of water from the dye-methylene blue (MB). The degradation rates of MB were 76% and 92%, respectively, under 120 min of simulated sunlight exposure. The efficient separation and transport of photogenerated carriers, as well as the large specific surface area, meant that the CaIn_2_O_4_-In_2_O_3_ composites were characterized by high photocatalytic efficiency. A. Ahmad et al. [116] by the triaxial electrospinning method produced TiO_2_ with a structure of nanofiber-in-nanotube (rutile-anatase), with which the photodegradation was carried out for 88.1% of the Sandalfix N. Blue with a 240 min irradiation time.

The diversity of available variations of the electrospinning process makes it possible to obtain MOS with high photocatalytic activity; however, further research is needed to explore the mechanism of this phenomenon.

The growing demand for green energy motivates researchers to look for materials and solutions that can increase the efficiency of existing renewable energy sources (RES), especially photovoltaic cells. So far, the many works that have presented the possibility of using 1D MOS in the construction of modern solar cells mainly focused on the use of TiO_2_, ZnO and SnO_2_ [117,118,119,120,121,122,123,124,125].

**Figure 11:**
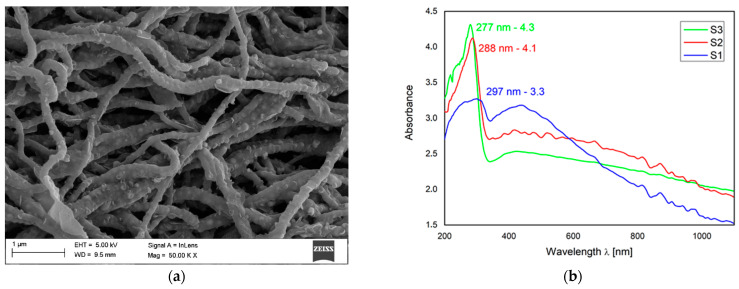
(**a**) SEM image of SnO_2_ nanowires calcined in 500 °C (Reprinted with kind permission from Springer [24]); UV-VIS: (**b**) absorption spectrum of SnO_2_ nanowires calcined in 500 °C (Reprinted with kind permission from Nature [126]).

**Figure 12:**
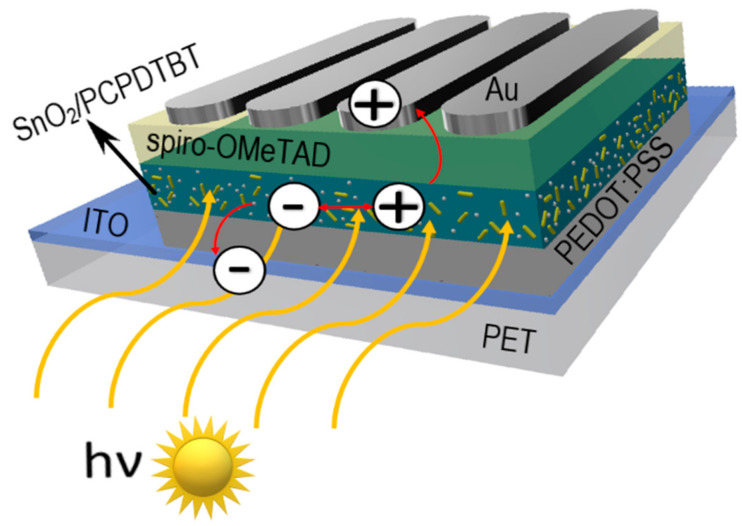
Schematic representation of multilayer flexible photovoltaic architecture manufactured in Department of Engineering Materials and Biomaterials [Poster at the 5th China (Shanghai) International Invention & Innovation Expo: “Innovative flexible solid-state solar cell with a hybrid layered architecture”].

Favorable optoelectronic properties of crystalline-amorphous hybrid SnO_2_ nanowires are suggested by W. Matysiak et al. [24] to be used in in modern flexible photovoltaic cells (Table 5, Figure 11). The research group, to which the Authors belong, was awarded a silver medal at the 5th China (Shanghai) International Invention & Innovation Expo in 2021 for the invention “Innovative flexible solid-state solar cell with a hybrid layered architecture”, for which the construction of which SnO_2_ nanowires were used (Figure 12).

M. Yang et al. in their publication [127] described the effect of graphene oxide (GO) admixture in hybrid SnO_2_/TiO_2_ nanofibers on the efficiency of dye-based solar cells (DSCs) constructed with their participation. DSCs along with GO-SnO_2_/TiO_2_ as the working electrode were analyzed for efficiency and the following photovoltaic parameters: short-circuit current density, open-circuit voltage and fill factor, which were respectively 11.19 mA/cm^2^, 0.72 V and 0.67. It was found that the solar-to-electric energy conversion efficiency of GO/SnO_2_/TiO_2_ as a photoanode-based device was 5.41%.

Therefore, it is worthwhile to pay attention to the application of MOS in the construction of next-generation photovoltaic cells, as they may provide a solution to the problem of low efficiency of dye-based cells.

### 3.2. Electrospun Metal Oxides 1D Nanostructures in Gas Sensors

The most widely studied application of one-dimensional metal oxide-based nanostructures are sensors for gases such as methanol, ethanol, acetone, formaldehyde, xylene and other volatile organic compounds that are highly toxic and dangerous to human health and even life [12,128].

Gas sensors based on semiconductor metal oxides are widely used in many areas, including chemical pollution control in air and rooms, alarms to detect the threat of poisonous substances and even medical diagnostics performed on the basis of a patient’s breath. The popularity of these types of sensors is due to their high sensitivity, low cost and ease of manufacture, as well as their compatibility with modern electronic devices [129,130,131,132,133,134]. 

The mechanism of gas detection by these MOS can be explained by the fact that the conductivity of the materials is changed by the chemical interaction between the gas and the surface of the nanostructure on which oxygen is adsorbed. Oxygen (O_2_) molecules are adsorbed on the nanofiber/nanowire surface in air and then they capture electrons from the conductivity band of the oxide so that chemisorbed oxygen ions (O_2_^−^) are generated and the formation of a barrier layer at a certain depth of the oxide structure is initiated. When the nanostructures are exposed to gas at an appropriate temperature, the gas reacts with the surface oxygen species and the width of the barrier layer decreases. As a result, the carrier concentration will increase, which ultimately increases the conductivity of the nanofibers/nanowires [135,136,137]. 

**Figure 13:**
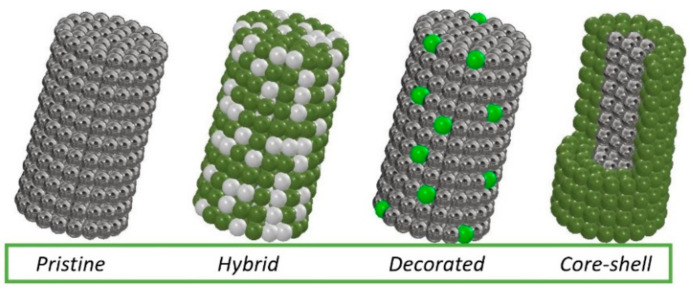
Types of morphology of the most commonly produced 1D nanostructures.

Many scientific reports indicate that the detection of hazardous substances by sensors based on electrospun MOS still needs to be developed—obtaining sensors with a lower substance detection threshold and shorter device response and reaction times. Improvement of these properties can be achieved by admixing with metallic nanoparticles, other MOS and carbon materials, which will affect the conductivity of the MOS. The combination of different materials produces local p-n, n-n or p-p nanojunctions. It is the heterojunctions generated from different materials that directly affect the substance detection mechanism. Several typical morphologies of MOS-based heterostructured materials are most commonly reported in the literature (Figure 13). In addition to non-admixed 1D MOS, hybrid structures consisting of both MOS and admixed crystallites simultaneously stand out. MOS nanowires decorated with nanoparticles or other forms of admixture are another interesting variation. There are also structures with core-shell morphology in which MOS can be either covered or surrounded by other material. 

One of the most commonly used MOS as detector anode is tin dioxide, which is characterized by an energy gap width of about 3.6 kV and simultaneous optical transparency and electrical conductivity [136,138,139,140,141,142]. Indium oxide exhibiting similar properties to tin oxide is also increasingly used. These materials are often combined with each other and also admixed with other oxides such as TiO_2_, ZnO, CuO and NiO (Table 6). The authors of this paper have established a collaboration with the Department of Optoelectronics, which is equipped with laboratories capable of gas detection measurements. Electrospun SnO_2_ and In_2_O_3_ nanowires fabricated in the Department of Engineering Materials and Biomaterials will be plotted on the IDT and tested to detect gases such as NH_3_, NO_2_, CO_2_ and H_2_. 

**Figure 14:**
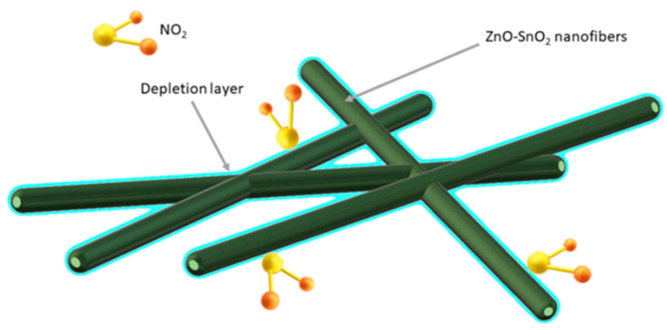
Scheme of hollow structure of gas adsorption and depletion layer for ZnO-SnO_2_ composite [143].

Bai et al. [143] demonstrated that the porous, coreless structure of ZnO-SnO_2_ nanowires is ideal for detecting very low concentrations (0.023 ppm) of toxic NO_2_. In addition, good detection properties of NO_2_ promotes the formation of an n-n heterojunction at the phase boundary of ZnO and SnO_2_, which results in the formation of an additional barrier layer (Figure 14).

Zhang et al. [144] observed that the response of sensors in acetone-containing environment can be improved by using heterojunction nanotubes of WO_3_-SnO_2_ and admixing it with Pd catalyst. Studies of the sensory properties of the material showed that the addition of Pd increased the response of Pd-WO_3_-SnO_2_ sensor more than double the response obtained from WO_3_-SnO_2_ sensor in contact with 100 ppm acetone. In addition, the selectivity for detecting acetone in the presence of other gases such as toluene, ammonia, nitrous oxide and pentane was significantly improved. 

**Figure 15:**
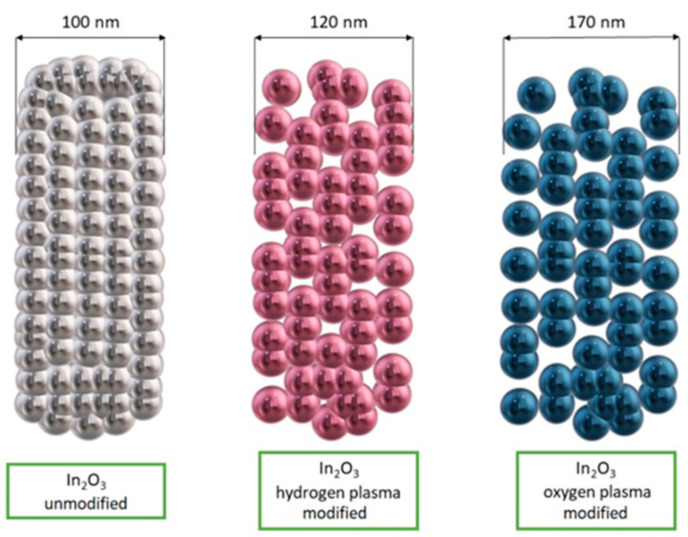
Scheme of In_2_O_3_ nanofibers morphology before and after surface modification [145].

Du et al. [145] fabricated In_2_O_3_ nanofibers with a traditional electrospinning method and then they subjected them to surface modification using low-temperature oxygen and hydrogen radiofrequency plasma. The nanofibers were placed in a plasma reactor chamber and surface modification was performed by increasing the number of pores and channels in the nanofibers (Figure 15). This mechanism enabled more oxygen to be adsorbed on the surface of the indium oxide nanostructures, leading to increased response values and improved selectivity for detecting acetone in the presence of interfering gases such as ethanol, methanol, formaldehyde, benzene, ammonia and nitrogen dioxide.

**Table 6:** Selected 1D MOS and their sensing properties.

Material Type	Polymer	Precursor	Solvent	Calcination		Gas	Conc [ppm]	Response/Recovery Time [s]	Ref.
				Time [h]	Temp [°C]				
ZnO-SnO_2_	PVP	SnCl_2_·2H_2_O, Zn(AC)_2_⋅2H_2_O	DMF, EtOH	3	600	Toluene	1-300	6–11/12–23	[146]
NiO-SnO_2_	PVP	SnCl_2_·2H_2_O, NiCl_2_·6H_2_O	DMF, EtOH	5	600	Toluene	50	11.2/4	[147]
CuO-SnO_2_	PVA	SnCl_2_·2H_2_O, CuCl_2_·2H_2_O	DMF, EtOH	4	600	H_2_S	10	1/10	[148]
CeO_2_-SnO_2_	PVP	SnCl_2_·2H_2_O, Ce(NO_3_)_3_·6H_2_O	DMF, EtOH	3	600	EtOH	200	8–10/11–30	[149]
W_2_O_3_-SnO_2_	PVP	SnCl_2_·2H_2_O/ (NH_4_)_6_H_2_W_12_O_40_·xH_2_O	DMF, EtOH	1	600	EtOH	10	18.5/282	[150]
Fe_2_O_3_-In_2_O_3_	PVP	In(NO_3_)_3_·4.5H_2_O, Fe(NO_3_)_3_·9H_2_O	DMF, EtOH	2	550	Formaldehyde	100	5/25	[151]
WO_3_-In_2_O_3_	PVP	In(NO_3_)_3_·4.5H_2_O, WCl_6_	DMF, EtOH, AcOH	2	500	Acetone	25	6/64	[152]
CuO-In_2_O_3_	PVP	In(NO_3_)_3_·*x*H_2_O, Cu(NO_3_)_2_·*x*H_2_O	DMF	2	600	H_2_S	5	4–30/incomplete recovery	[153]
SnO_2_-In_2_O_3_	PVP	In(NO_3_)_3_·4.5H_2_O, SnCl_2_·2H_2_O	DMF, EtOH	2	600	Formaldehyde	0.5-50	~20/40	[154]
In_2_O_3_ (RF plasma modified)	PVP	In(NO_3_)_3_·4.5H_2_O	DMF, EtOH	3	550	Acetone	10	18–23/55–92	[145]
La_2_O_3_-In_2_O_3_	PVP	In(NO_3_)_3_·*x*H_2_O, La(NO_3_)_3_·xH_2_O	DMF, EtOH, mineral oil	2	550	Formaldehyde	50	3/19	[155]
In_2_O_3_	PVP	In(NO_3_)_3_∙4.5 H_2_O	DMF	2	800	NO_2_	5	200/1000	[156]

The above considerations indicate that electrospun one-dimensional MOS plays a key role in the construction of gas sensors, thus contributing to their development and improving work and life safety in environments exposed to hazardous gases.

### 3.3. Electrospun Metal Oxides 1D Nanostructures in Other Applications

Supercapacitors and lithium-ion batteries (LIBs) are other devices for which one-dimensional MOS nanostructures can be used. With the rapid progress of civilization and industrialization, there is a growing need for methods, materials and devices to store large amounts of energy [157]. One solution to meet these needs is the development of LIBs with high performance, which is primarily dependent on the performance of the battery’s most important component, the anode. The currently used anode material in the form of graphite is currently no longer able to meet the needs of high energy storage capacity due to its low capacity and low efficiency. Therefore, the search and research of new electrode materials is of great importance for the current demand for high performance LIBs [30,158]. Recently, semiconductor nanomaterials such as ZnO, NiO, SnO_2_ lub TiO_2_ nanotubes and nanowires have been of particular interest for 1D, along with heterojunctions formed by combining these materials with carbon materials [159,160,161,162,163,164]. The advantage of using one-dimensional nanomaterials for anodes in LIBs is the much less frequently observed agglomeration of the material than in the case of nanoparticles, which positively affects the electrochemical performance of the battery, and this fact was confirmed in a study by C. K. Chan et al. [165] based on the analysis of a battery based on Si nanowires. 

**Figure 16:**
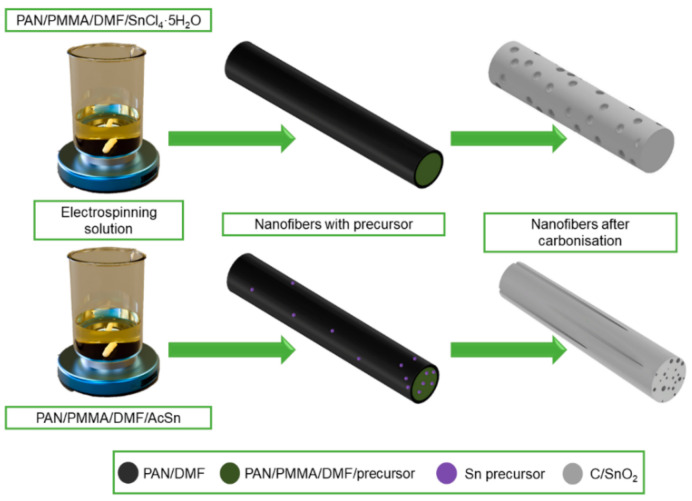
Scheme of the manufacturing process of C/SnO_2_ nanofibers with a different morphology.

J. Zhu et al. [163] pointed out the high application potential of electrospun ZnO-SnO_2_ nanofibers as anode material in lithium-ion batteries. It was observed that due to the heterogeneous mesoporous electrode structure based on ZnO-SnO_2_ nanofibers, they provide excellent performance and reversible capacity at a relatively low cost and with high process repeatability. D. Lei et al. in their work [166] showed that GeO_2_-SnO_2_ composite nanofibers with high porosity prepared by the solution electrospinning method have high specific capacitance and good cycling performance, which is mainly due to the porous one-dimensional nanostructure, which can shorten the transport pathway and provide trapping of electrolyte ions to meet the requirements of fast charging and discharging reactions. J. Guo in [167] described the effect of pore distribution on the capacitance of two types of porous C/SnO_2_ nanofibers produced by electrospinning from solutions based on different precursors, i.e., using tin chloride, the fibers with spherical pores were obtained, while the pores in the form of channels were obtained from acetate (Figure 16). On the basis of a galvanostatic charge-discharge test, it was found that multichannel C/SnO_2_ nanofibers with a large specific surface area (34.97 m^2^/g) achieve better charging performance than spherical pore nanofibers and show a more stable capacity retention of about 90% after 50 cycles.

The use of SnO_2_-ZnO nanofibers in energy storage was presented in the work [164] of J. Zhang et al. The study showed that by using the spinning solution parameters, it is possible to control the morphology and obtain hollow nanotubes, which exhibited good capacity stability in an electrochemical test. In addition, it was observed that the polypyrrole (PPy) polymer coating of SnO_2_-ZnO nanotubes has made it possible to maintain a high capacity of 626.1 mA hg^−1^ at 0.2 °C for 100 cycles, and cycle stability has also been improved.

Thus, the electrospinning method with subsequent calcination enables precise control of the electrochemical properties of the fabricated one-dimensional MOS-based nanostructures, thus providing a chance to solve the problem of non-compliant LIBs. 

Due to their unique optical, electrical and magnetic properties, they are used in modern devices such as field-effect transistors (FETs) and microwave absorption materials. X. Zhu et al. presented [168] a method to fabricate high-performance field-effect transistors based on electrospun In_2_O_3_ nanofibers admixed with Al, Ga and Cr. The devices showed optimal performance at a 10% molar concentration of admixing material (Al, Cr and Ga): low and positive gate-source voltage V_GS_ (<6.0 V), a high ratio of the transistor on current to transistor off current I_on_/I_off_ (~108), high saturation current (~10–4 A) and carrier mobility on the level of ~2.0 cm^2^/V^−1^s^−1^.

H. Zhang et al. [111] demonstrated that the use of polymorphic anatase-rutile TiO_2_ nanofibers to build FET showed better transistor characteristics because of a strong synergistic effect compared with pure anatase and rutile TiO_2_ nanofibers. BioFET created by S. Veeralingam and S. Badhulik [97] based on β–Bi_2_O_3_ nanofibers for the detection of serotonin exhibited sensitivity of 51.64 μA/nM over a range of 10 nM^−1^ μM and a limit of detection of 0.29 nM. Moreover, it maintained excellent sensitivity, stability and reproducibility with a rapid response time of 0.8 s. Using the electrospinning method, K.C.S. Reddy et al. [169] created a self-powered NiO-p/Si-n based ultraviolet photodetector which exhibited a high responsivity of 9.1 mA W^−1^ at zero bias with a fast photoresponse of less than 0.4 s. X. Huang et al. [102] observed that electrospun bead-like Co-ZnO nanostructures present ferromagnetic properties and an excellent electromagnetic loss performance—the effective microwave absorption of bandwidth with reflection loss less than −10 dB was 11.6 GHz.

For years, medicine has been a priority discipline in which new solutions and biomaterials are constantly being sought. Looking at the disease problems that affect mankind today, the most rapidly developing areas of medicine include cancer therapies, drug delivery, biosensors, medical imaging and tissue engineering. Due to the unsatisfactory properties of conventional biomaterials, it is necessary to search for new material solutions. Production of one-dimensional nanomaterials with controlled dimensions, arrangement of structures with respect to each other or porosity creates many possibilities of using their unique properties for therapeutic purposes. Ceramic nanomaterials, which are based on inert simple oxides, may seem to be a possible solution for some health problems. The most commonly used one-dimensional MOS include TiO_2_, due to its non-toxicity, environmental friendliness as well as good chemical stability and high corrosion resistance [170,171]. 

One of many interesting examples of work on the above issue is that presented by I.H.M. Aly et al. [172], who used electrospun TiO_2_ nanofibers as an admixture to a bioceramic composite based on wollastonite for bone tissue regeneration, which significantly improved the mechanical properties of the composite while not affecting the bioactivity in any way, and proves that this type of material is worth considering and researching for applications in medicine. Mesh with TiO_2_ nanofibers may also find applications in tissue engineering, as studies have shown that it provides an osteogenic environment—increasing osteoblast production and differentiation [173]. S. Chen et al. confirmed the possibility of using hydrothermal treated nanofibers as delivery systems for the antibiotic tetracycline hydrochloride, whereby nanofibers showed high bactericidal activity against *E. coli* and *S. aureus* [174]. N.C. Bezir et al. demonstrated that TiO_2_ and Ag/TiO_2_ nanofibers show beneficial antibacterial properties based on measured inhibition zones diameters of *S. aureus*culture plates [175]. Effective inhibition of *B. subtilis* and *B. cereus* through TiO_2_/GO/CA nanofibers was observed by L. Jia et al. [176]. TiO_2_ in the form of electrospun one-dimensional nanostructures also shows promising results in promoting apoptosis of cancer cells, e.g., cervical cancer [177]. Other applications of 1D ceramic nanomaterials in medicine include the use of oleic acid-coated ZnO nanowires to fabricate hydrophobic polyvinylidene fluoride (PVDF) membranes, whose self-cleaning properties can be used to construct surgical devices and instruments or artificial blood vessels [178]. ZnO nanofibers, similarly to TiO_2_ nanofibers, are characterized by tremendous antibacterial activity in *S. aureus and E. coli* utilization [179,180].

## 4. Summary and Outlook

This review is an attempt to summarize electrospun one-dimensional MOS nanostructures fabrication, state-of-art and application possibilities.

The last quarter century has witnessed a dynamic growth of interest in one-dimensional metal oxide-based nanostructures, which include nanofibers, nanowires, nanorods, nanotubes, etc., in both academia and industry. This is evidenced by the ever-increasing number of publications and research works undertaken in the field of fabrication by various methods, analyses of chemical and physical properties and application potential of 1D MOS. The continuous development of methods for the fabrication of these nanostructures has led researchers to combine sol-gel and electrospinning methods through which, without the need for complex methodology, it is possible to obtain nanomaterials of the desired structure and properties on a laboratory and industrial scale. This method allows for precise control of morphology, structure and consequently optical, electrical and magnetic properties. The key to the manufacturing of 1D nanostructures with the desired properties is the production of solutions with a viscosity that allows spinning and the use of appropriate process parameters, which must be self-adjusted, because even spinning the same material, but on a different type of equipment, may require different parameters. This creates wide application possibilities for 1D MOS in the construction of modern opto-electronic devices, gas sensors, flexible devices, biomedical electronics and in photocatalytic purification of aqueous environments.

Despite the many advantages of electrospun 1D MOS, there are also some challenges associated with the properties of these materials. In spite of a variety of available nanomaterials, it is still a challenge to improve the photocatalytic performance of electrospun MOS by carefully selecting suitable co-catalysts in suitable concentrations for doping and heterojunction formation. Furthermore, future investigations are needed to design MOS photocatalysts for a visible light driven heterogeneous photocatalysis. It is also worthwhile to pay special attention to the application of such materials in renewable energy sources, in particular the conversion of solar mechanical energy to electrical energy, as these materials may represent the future of powering personal electronics. However, for this to happen, it is necessary to design systems with both high flexibility and energy conversion efficiency. Despite advanced research on electrospun MOS sensing properties, new admixing materials and gas sensing mechanisms are still being explored and developed to provide sensors with the highest possible sensitivity and fastest possible response.

Studies analyzing the impact of one-dimensional MOS nanostructures on the environment and human health should also be undertaken, as these are key factors in determining the potential for these materials to enter everyday use.

The multitude of benefits offered by 1D MOS fabricated by electrospinning should prompt researchers to further explore this area of nanotechnology.

## Figures and Tables

**Table 2:** Selected popular 1D metal oxides with examples of reagents used for their fabrication.

Material	Polymer	Precursor	Calcination Temperature	Ref.
TiO_2_	PVP	Ti(OCH(CH_3_)_2_)_4_	450	[85]
TiO_2_	PVP	C_16_H_36_O_4_Ti	500	[86]
ZnO	PVA	Zn(CH_3_CO_2_)_2_·2H_2_O	400/500/600	[65] *
ZnO	PVP	Zn(CH_3_CO_2_)_2_·2H_2_O	350–600	[87]
SiO_2_	PVP	Si(OC_2_H_5_)_4_	600	[22]
SiO_2_	PVA	Si(OC_2_H_5_)_4_	600	[88]
WO_3_	PVA	(NH_4_)_6_H_2_W_12_O_40_H_2_O	400/500/600/700	[89]
WO_3_	PVP	WCl_6_	450–600	[90]
CuO	PVP	Cu(CH_3_COO)_2_	500/600/700/800/900/1000	[91] *
CuO	PVP	Cu(NO_3_)_2_∙3H_2_O	-	[92]
Fe_2_O_3_	PVA	Fe(NO_3_)_3_·9H_2_O	800	[93]
Fe_2_O_3_	PVP	Fe(CH_3_COO)_2_	500/600/700/800/900/1000	[94]*
SnO_2_	PVA	SnCl_2_·2H_2_O	300/500/700	[95]
SnO_2_	PVP	SnCl_2_·2H_2_O	500/600	In press *
Bi_2_O_3_	PAN	Bi(NO_3_)_3_	500/550/600	[96]
Bi_2_O_3_	PVP	Bi (NO_3_)_3_.5H_2_O	450	[97]
In_2_O_3_	PVP	In(NO_3_)_3_·4.5H_2_O	600	[18]
In_2_O_3_	PVP	In(NO_3_)_3_·*x*H_2_O	600	[98]

* Polish scientific units.

**Table 3:** Optical properties of selected 1D metal oxides prepared via electrospinning.

MOS	TiO_2_	ZnO	SiO_2_	SnO_2_	Bi_2_O_3_	In_2_O_3_
Direct band gap [eV]	2.91–2.94	3.32–3.36	3.93–3.97	3.30–3.58	2.48–2.72	2.92–3.34
Ref.	[86]	[65]	[11]	[108]	[109]	[23,110]

**Table 4:** Optical properties of TiO_2_ nanowires and ZnO nanowires after calcination at 400 °C [own study].

Material	Calcination Temperature [°C]	Max. Absorbance	Wavelength [nm]	Eg [eV]
TiO_2_ nanowires	400	2.42	248	3.73
	500	2.34		3.83
	600	2.26		3.88
ZnO nanowires	400	2.94	346	3.36
	500	3.38		3.34
	600	3.43		3.32

**Table 5:** Refractive index and dielectric permittivity obtained for the electrospun 1D SnO_2_ nanomaterials [24].

Parameter	SnO_2_ Nanowires		
	Type of Spinning Solutions		
	Type 1	Type 2	Type 1
refractive index (n)	1.51	1.52	1.51
complex dielectric permeability (ε)	2.28	2.30	2.28
Energy band gap (E_g_)	3.3	3.8	3.9

## Data Availability

Not applicable.
